# Partial adoption of ‘minimal core curriculum’ in undergraduate teaching of family medicine: A cross-sectional study among Central and South-Eastern European medical schools

**DOI:** 10.1080/13814788.2018.1464555

**Published:** 2018-05-05

**Authors:** Irena Zakarija-Grković, Venija Cerovečki, Davorka Vrdoljak

**Affiliations:** a Department of Family Medicine, University of Split School of Medicine, Split, Croatia;; b Department of Family Medicine, University of Zagreb School of Medicine, Zagreb, Croatia

**Keywords:** Medical education, cross-sectional designs

## Abstract

**Background:** In 2011, Tandeter et al. published a list of 15 themes, based on a Delphi survey among representatives of the European Academy of Teachers in General Practice and Family Medicine (EURACT), and suggested this be the ‘minimal core curriculum’ (MCC) for undergraduate education in family medicine.

**Objectives:** To determine: (1) if medical schools in the former Yugoslavia region are familiar with the MCC; and (2) to what degree it is being taught to medical students.

**Methods:** In July 2015, a questionnaire was distributed to 19 medical schools in the former Yugoslavia region. A copy of the description of the curriculum for GP/FM was requested from participants. Two researchers conducted content analysis of the curricula according to the 15 predefined MCC themes, independently.

**Results:** Thirteen (68%) medical schools responded. Of these, 10 (77%) stated that they were familiar with the MCC. Not a single institution encompassed all 15 MCC themes. The number of themes included by individual medical schools ranged from 6/15 (40%) to 13/15 (87%).The following themes were covered by 12 of 13 (92%) medical schools: Introduction to GP/FM; communication skills; prevention and health promotion; and management of chronic diseases. The three themes most poorly covered were: consulting skills (5/13), management of diseases at an early, undifferentiated stage (2/13) and decision-making based on prevalence and incidence (1/13).

**Conclusion:** Despite familiarity with EURACT’s MCC among medical schools in the former Yugoslavia region, significant variation in curricula content exists, and no curriculum covered all MCC themes.

KEY MESSAGES

- Most medical schools in the former Yugoslavia region are familiar with the ‘minimal core curriculum’ (MCC) for family medicine.
- The family medicine curricula of these medical schools vary considerably in the degree of compliance with the MCC.
- There is a need for revision of curricula in line with current recommendations and further clarification of MCC themes.

## Introduction

An agreed-upon minimal undergraduate curriculum content is necessary for the teaching of every discipline to ensure students gain insight into the specific attributes of a particular specialty as well as to enable standardization of teaching. The latter facilitates communication among colleagues, sharing of teaching resources and exchange of students and staff. A group of experts, considered leaders in their field, usually defines these minimal requirements through a form of consensus.

General practice/family medicine (GP/FM), as an academic discipline, has undergone this process, with various societies, in different countries, undertaking the task of defining a basic curriculum for teaching undergraduate medical students [1–4]. Education of medical students is nowadays well recognized as an essential basis for the further development of GP/FM in many countries of Central and Eastern Europe. Efforts have been made in those countries to improve undergraduate education further, both through didactic teaching of medical students, as well as through real-life practical work with experienced GPs as mentors [5].

In 2011, the Basic Medical Education Committee of the European Academy of Teachers in General Practice and Family Medicine (EURACT) published a list of 15 themes, compiled using the Delphi method among 40 EURACT Council members, considered minimal core curriculum (MCC) for a clerkship of very short duration [6]. Optimistically, this list would be useful for institutions developing a new primary care programme as well as serve as a basis for the teaching of GP/FM. To the best of our knowledge, implementation of this suggested minimal core content has not been evaluated; hence, the aims of this study were to determine if medical schools in the region of the former Yugoslavia are first, familiar with the MCC, and second, to what degree the 15 themes are incorporated in undergraduate GP/FM curricula.

## Methods

### Study design

This study was part of a more extensive cross-sectional study looking at the organization of family medicine teaching in countries of the former Yugoslavia.

### Ethics

Approval for this study was received from the University of Split, School of Medicine Ethics Committee (No. 2181-198-03-04-15-0004).

### Selection of medical schools

We chose medical schools that are located in countries of the former Yugoslavia for ease of communication and access to data. Of 21 medical schools, 19 with valid addresses were sent a semi-structured questionnaire directed to the heads of the departments of GP/FM or the vice-dean for teaching, if no formal department existed. The 45-item questionnaires were dispatched in July 2015, with two follow-up reminders sent electronically to non-responders at monthly intervals.

### Data

In the questionnaire, participants were asked whether they were familiar with the MCC in family medicine, to which they could reply ‘yes’ or ‘no.’ All medical schools were asked to send a copy of the official family medicine curriculum for undergraduate students, along with a detailed description of the course content to the study authors.

For the current study, only results relating to familiarity with the MCC and content analysis of the teaching programmes are shown; the remaining results on the organization of undergraduate teaching of family medicine (44 items) will be published elsewhere.

### Analysis

Each curriculum was studied in detail by two independent assessors (IZG and DV) who searched for the 15 themes defined by Tandeter et al. Each theme was graded as 1 = included, 2 = omitted and 3 = partially included. Partial scores were given to teaching units, which included components of the themes but were not explicitly dedicated to them; e.g., if management of symptoms such as abdominal pain or fever were listed in the curriculum, then a partial score was given for theme three (management of diseases at early, undifferentiated stage). Scoring sheets were sent independently to the third team member (VC) who resolved any discrepancies that arose.

Data from questionnaires and programme content analysis outcomes were entered into Excel spreadsheets. Descriptive statistics were used to analyse the data using MedCalc statistical software, version 17.1 [7].

## Results

### Participants

Thirteen of 19 medical schools responded (response 68%), based in the following cities: Belgrade (Serbia), Foča, Mostar, Sarajevo, Tuzla (Bosnia and Herzegovina), Ljubljana, Maribor (Slovenia), Osijek, Rijeka, Split, Zagreb (Croatia), Podgorica (Montenegro) and Skopje (former Yugoslav Republic of Macedonia).

### Familiarity with MCC

Ten of 13 medical schools (77%) stated that they were familiar with the ‘minimal core curriculum’.

### Inclusion of MCC in teaching

There was considerable variation in the number of themes included by individual medical schools in our sample, ranging from 6 (40%) to 13 (87%), with not a single institution encompassing all 15 themes (Table 1).

**Table 1.** Inclusion of ‘minimal core curriculum’ themes in GP/FM curricula in 13 medical schools of the former Yugoslavia region.

Medical school	Number of included themes (15 themes in total)
Ljubljana, Osijek	13
Maribor, Sarajevo	12
Rijeka	11
Skopje, Zagreb	10
Split, Tuzla	9
Podgorica	8
Belgrade, Foča	7
Mostar	6

The following themes were covered by 12 of 13 (92%) medical schools: introduction to GP/FM; communication skills; prevention and health promotion; and management of chronic diseases. The three themes most poorly covered were: consulting skills (5/13), management of diseases at an early, undifferentiated stage (2/13), and decision-making based on prevalence and incidence (1/13) (Table 2).

**Table 2.** Frequency of inclusion of ‘minimal core curriculum’ themes among 13 participating medical schools in the former Yugoslavia region.

Theme [6]	Medical schools (*n* = 13) *n* (%)
Introduction to GP/FM as a specific medical discipline. Principles of FM.	12 (92)
Communication skills: with patient, with relatives, with ‘difficult’ patients.	12 (92)
Prevention and health promotion, patient education.	12 (92)
Chronic care, management of chronic diseases and health problems.	12 (92)
The specific characteristics of healthcare in FM.	11 (85)
Most common presenting symptoms in family practice.	11 (85)
Interface of primary and secondary care: referrals, gate keeping, advocacy.	10 (77)
Management of multiple health problems, identifying priorities.	9 (69)
Patient-centeredness.	9 (69)
Community orientation; community-centred care; community needs assessment.	8 (62)
The family as a source of disease and resource of care; genograms; life cycle.	7 (54)
Holistic approach. Biopsychosocial model.	6 (46)
Consulting skills—stages of a consultation.	5 (39)
Management of diseases at early undifferentiated stage. Dealing with uncertainty.	2 (15)
Decision-making based on prevalence and incidence of target.	1 (8)

## Discussion

### Main findings

Over three-quarters of medical schools in the region of the former Yugoslavia stated that they were familiar with the MCC. Despite alleged widespread knowledge of MCC, not a single participating school implemented all core content in its GP/FM undergraduate programme. The number of themes included by individual medical schools ranged from 6/15 to 13/15.

### Strengths and limitations

To the best of our knowledge, this is the first study to report on European medical schools’ awareness of EURACT’s recommended MCC. It is also the first to evaluate, using three independent assessors, the degree of implementation of MCC in GP/FM undergraduate teaching.

Our analysis of family medicine curricula was limited by the amount of detail available in the course programmes. It is likely that more themes were covered than indicated by the title and description of the teaching units; therefore, our assessment may be an under-representation of the themes covered. Another limitation is possible participant bias, given that only 13 of 19 medical schools in the region responded. Survey respondents are usually those who are more ambitious, knowledgeable and have something to show, making our findings not necessarily representative of the region as a whole. Finally, our results are now two years old; hence, may not represent the current situation in participating medical schools. We intend to repeat our study shortly to evaluate whether involvement in this research project has led to an increase in MCC implementation.

### Interpretation of study results

We consider the fact that a majority of medical schools stated they were familiar with the MCC a positively surprising finding, given the limited influence of EURACT’s recommendations on individual country teaching programmes for undergraduate students in GP/FM. Despite this awareness, none of the abovementioned medical schools had incorporated all 15 core themes.

The most poorly covered themes were ‘consulting skills—stages of a consultation,’ ‘management of diseases at an early, undifferentiated stage’ and ‘decision-making based on prevalence and incidence of target.’ Interestingly, all three themes were ranked by EURACT council members among the top 10 most important themes, with ‘consulting skills’ ranked ninth, ‘management of disease at an early stage,’ third and ‘decision-making based on prevalence and incidence of target,’ sixth. So why were they not included by medical schools aware of the MCC? Non-adherence may be a sign that individual themes were seen as less important, difficult to teach or that the listed theme was simply unclear, i.e., poorly defined. As stated by Tandeter et al., ‘The North American curriculum recommendations go into substantial detail regarding each theme’ [6]. Uptake of the MCC would possibly be higher if greater detail was provided for each theme by EURACT, especially for the topic most poorly covered by our participating medical schools, ‘decision-making based on prevalence and incidence of target’. This would also enable assessment that is more precise during future audits.

Considerable variability is seen between the top and bottom ranking schools, with those at the top including double the number of core themes in their undergraduate curricula as those on the bottom. A closer study of the individual schools reveals that those who were ranked higher had established departments of GP/FM (founded in the 1990s), whereas the medical schools with the lowest number of included themes either did not have a department of GP/FM at the time or it had been established in the last 15 years. Possibly, countries where GP/FM has a more extended tradition, and hence stronger position, teachers of family medicine are more likely to be involved in EURACT activities or have more capacity to influence the curriculum.

### Implications for education, policy and research

Given the incomplete uptake of MCC content in participating medical schools, EURACT may need to explore this phenomenon to understand better why so few teach curriculum that closely matches the MCC, given that it has been several years since the MCC for family medicine was developed and widely published [6,8]. In our study, three medical schools were not aware of MCC, even though participating countries are involved in EURACT activities either as representatives or active participants of teaching courses, which raises the question of dissemination strategies used in informing teachers of family medicine in Europe and how this can be improved. In a study by Cochella et al. [9], 92% of clerkship directors were aware of the US National Clerkship Curriculum for Family Medicine (NCC), whereas in our sample only 77% of participants gave a positive response. A likely reason for the high level of awareness of NCC in the US, is the availability of tools to support implementation of NCC, including an NCC website containing the NCC objectives, peer-reviewed sample curricula, information on educational methods, assessment strategies and faculty development. In their study, Cochella et al. found that clerkship directors placed greatest value on materials that could be downloaded and adapted to individual clerkships (undergraduate rotations in GP/FM). If EURACT were able to provide similar tools, more widespread implementation of MCC would probably ensue, although this would ideally need to be preceded by a needs assessment of curriculum directors. An important document entitled ‘Framework for continuing educational development of trainers in general practice/family medicine in Europe’ was published by EURACT in 2012 and it may help achieve this goal [9]. This guides the development of GP/FM educators. Also, EURACT regularly organizes workshops and courses for trainers/educators of GP/FM from the region. Different GP/FM topics are taught and discussed. Future courses could focus on the MCC and ways to implement it.

The MCC is a consensual list of 15 core themes to be covered during an undergraduate course in family medicine of at least one-week duration, defined and accepted by leading European teachers in FM. Therefore, our aim should be for it to be approved and implemented by all European medical schools. This would then provide us with a framework for preparing teaching materials, a focus for our exams in GP/FM and a standardized teaching programme that would aid in the collaboration and exchange of students and staff between institutions, as well as the sharing of teaching resources. The MCC may be used by medical educators not only for new programme development, as recommended by Tandeter et al., but also for the revision of existing programmes, targeting themes that are deficient [10,11]. Hopefully, the availability of data on the implementation of MCC in the region of the former Yugoslavia will prompt other European countries to evaluate their compliance with EURACT recommendations to enable comparison of results and encourage revision of curricula in line with MCC.

## Conclusion

Despite familiarity with EURACT’s MCC among countries of the former Yugoslavia, significant variation in curricula content was observed. No curriculum in our sample included all ‘core’ themes, suggesting a need for clarification of MCC themes and revision of GP/FM curricula.
